# Fetal Rapid Eye Movement Sleep Dysfunction as a Potential Early Indicator for NALCN‐Related CLIFAHDD Syndrome: A Case Report

**DOI:** 10.1002/pd.70096

**Published:** 2026-02-17

**Authors:** Fumiya Fukumori, Yoshiki Maeda, Kanemasa Maki, Toshiki Takenouchi, Kenjiro Kosaki, Hiromasa Funato, Eiji Kondo, Tomoaki Ikeda

**Affiliations:** ^1^ Department of Obstetrics and Gynecology Yokkaichi Municipal Hospital Yokkaichi Japan; ^2^ Department of Obstetrics and Gynecology Kuwana City Medical Center Kuwana Japan; ^3^ Department of Pediatrics Yokkaichi Municipal Hospital Yokkaichi Japan; ^4^ Department of Pediatric Neurology Okayama University Graduate School of Medicine Dentistry and Pharmaceutical Sciences Okayama Japan; ^5^ Center for Medical Genetics Keio University School of Medicine Tokyo Japan; ^6^ International Institute for Integrative Sleep Medicine (WPI‐IIIS) University of Tsukuba Tsukuba Japan; ^7^ Department of Obstetrics and Gynecology Mie University Hospital Tsu Japan

## Abstract

- What's already known about this topic?
  ◦ *NALCN* variants, especially gain‐of‐function variants, increase neuronal excitability and reduce REM sleep.
  ◦ CLIFAHDD syndrome, caused by *NALCN* gain‐of‐function variants, includes congenital contractures, hypotonia, and developmental delay.
- What does this study add?
  ◦ This report provides detailed prenatal documentation of absent REM‐associated cycling and extremely low fetal heart rate (FHR) variability in a fetus with CLIFAHDD syndrome.
  ◦ We demonstrated the absence of FHR variability and REM sleep cycling as potential early indicators of autonomic and sleep state dysregulation.
  ◦ REM sleep loss may contribute to neurodevelopmental abnormalities in affected fetuses.

The sodium leak channel nonselective (NALCN) protein is a critical regulator of neuronal excitability and sleep regulation. *NALCN* variants, particularly gain‐of‐function variants, reduce rapid eye movement (REM) sleep by increasing neuronal excitability in the deep mesencephalic nucleus [1]. Fetal sleep states, including REM and non‐REM sleep (NREMS), have been studied in animal models, demonstrating distinct developmental patterns during gestation. REM sleep predominates during late gestation, influencing breathing, swallowing, and muscle activity [2]. However, little is known about REM sleep regulation in human fetuses.

The dysfunction of NALCN and its subunits is strongly associated with congenital diseases, such as congenital contractures of the limbs and face, hypotonia, and developmental delay (CLIFAHDD), caused by a gain‐of‐function property, with excessively active *NALCN* and heterozygous variants [3]. CLIFAHDD syndrome (#OMIM: 616266) is a rare autosomal dominant disorder characterized by congenital contractures, hypotonia, respiratory distress, and developmental delay [3]. Given its rarity—estimated at fewer than one case per million [3]—prenatal diagnosis is challenging, and specific markers have not been established. Previous reports of CLIFAHDD cases described abnormal FHR, however, these findings were not explicitly interpreted in the context of fetal sleep or behavioral state regulation [4]. The present case expounds upon these observations by closely linking FHR abnormalities to disrupted fetal sleep‐state cycling.

A 32‐year‐old primigravida was referred to our facility at 19 weeks of gestation for perinatal care. At 30 weeks, the patient reported decreased fetal movements. Continuous cardiotocography showed a complete absence of variability and a prolonged monotonous acceleration with fetal movement without cycling (Figure 1a–b). The mean short‐term variability remained below 4 ms throughout the day between 30 and 34 weeks, markedly below normal reference values (7.79 ms; interquartile range: 6.35–9.66) [5]. Fetal ultrasonography and magnetic resonance imaging revealed mild ventriculomegaly (14 mm, left; 11 mm, right), which was regarded as a non‐specific finding. At 34 weeks and 3 days, an emergency caesarean section was performed due to premature rupture of membranes and breech presentation. The male neonate weighed 2100 g (+0.8 SD) and had low Apgar scores (2 and 3). He exhibited severe hypotonia, joint contractures, and no spontaneous movement. Genetic testing identified a de novo heterozygous NALCN variant, c.3970C>T (p.Leu1324Phe), classified as a likely pathogenic variant. Detailed results are provided in the Variant Validator Report (Table 1). The PP3 classification was not applied because most in silico tools are optimized for loss‐of‐function variant detection. The infant empirically received phenobarbital for subclinical seizure and was discharged for outpatient follow‐up care.

**FIGURE 1:**
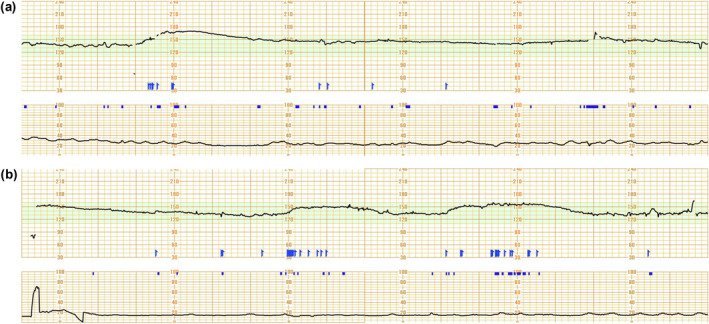
Fetal heart rate monitoring at (a) 30 + 1 weeks' gestation and (b) 34 + 4 weeks' gestation. No FHR variability was observed. A prolonged acceleration with fetal movement lasting several minutes with fetal movement occurred. Arrows indicate maternal awareness of fetal movements.

**TABLE 1:** Variant validator report.

Gene (name REFSEQ MANE select)	Known disease (OMIM)	Variant	ACMG classification	Criteria applied	Inheritance & zygosity	Interpretation	ClinVar accession number
NALCN (sodium leak channel, non‐selective) (NM_052867.4)	#611549	c.3970C>T; p.(Leu1324Phe); Chr13:g.101726998G>A (GRCh37)	Likely pathogenic	PS2 (de novo), PM2 (absent/rare in population databases)	de novo Heterozygous	Missense variant predicted damaging; associated with multiple joint contractures, neonatal seizures, cryptorchidism, and bilateral clubfoot	Not listed

This case highlights a unique prenatal manifestation of CLIFAHDD syndrome, specifically impaired REM sleep and absent FHR variability. REM sleep absence and FHR variability may support the disruption in sleep states and autonomic dysfunction in this disease, which might be reflected in other neurodevelopmental lesions or conditions, such as central nervous system injury and hypotonia. Research in animal models has shown that REM sleep is essential for cortical development and that its deprivation can lead to delayed neurodevelopment [6]. These findings suggest that in‐utero REM sleep disruption could be a contributing factor in the neurodevelopmental impairments seen in NALCN‐related disorders. However, given the widespread expression of NALCN, sleep‐state disruption might also reflect secondary neurodevelopmental abnormalities. The interplay between REM sleep, cortical maturation, and autonomic control is complex and still under investigation.

This case provides novel prenatal evidence linking NALCN variants with REM sleep disruption and autonomic irregularities in utero. The absence of FHR variability and sleep state cycling may serve as potential early indicators of NALCN‐related channelopathies, such as CLIFAHDD syndrome. These findings underscore the potential role of REM sleep in fetal brain development and point to the importance of incorporating fetal neurobehavioral monitoring into prenatal diagnostic strategies for rare genetic conditions.

## Funding

The authors have nothing to report.

## Ethics Statement

The Institutional Ethics Committee confirmed that a formal review was not required for a single anonymized case report; written informed consent was obtained.

## Consent

Informed consent was obtained from the patient for the use of their data.

## Conflicts of Interest

The authors declare no conflicts of interest.

## Data Availability

The data that support the findings of this study are available from the corresponding author upon reasonable request.
